# Enhancing Magnetic Hyperthermia Efficiency in Pd/Fe-Oxide Hybrid Nanoparticles through Mn-Doping

**DOI:** 10.1021/acsanm.4c05452

**Published:** 2024-12-04

**Authors:** Alexandra Maier, Qi Jia, Keshav Shukla, Achim Iulian Dugulan, Peter-Leon Hagedoorn, Rogier van Oossanen, Gerard van Rhoon, Antonia G. Denkova, Kristina Djanashvili

**Affiliations:** † Department of Biotechnology, Delft University of Technology, 2628 HZ Delft, The Netherlands; ‡ Department of Radiation Science and Technology, Delft University of Technology, 2629 JB Delft, The Netherlands; § Department of Radiotherapy, Erasmus MC Cancer Institute, University Medical Center, 3008 AE Rotterdam, The Netherlands

**Keywords:** hybrid nanoparticles, palladium, iron oxide, manganese doping, magnetic properties, hyperthermia/thermal ablation, MRI contrast

## Abstract

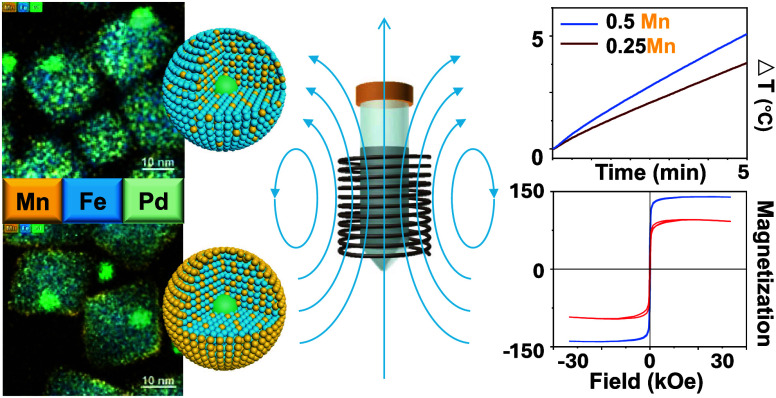

Multifunctional, biocompatible magnetic materials, such as iron oxide nanoparticles (IONPs), hold great potential for biomedical applications including diagnostics (e.g., MRI) and cancer therapy. In particular, they can play a crucial role in advancing cancer thermotherapy by generating heat when administered intratumorally and when exposed to an alternating magnetic field. This heat application is often combined with radio- (chemo)therapy and/or imaging. Consequently, the design of materials for such a multimodal approach requires hybrid nanoparticles that retain their magnetic properties while integrating additional functionalities. This work introduces synthesis and investigation of magnetically enhanced nanoparticles with a palladium core (envisioned for future radiolabeling with therapeutic ^103^Pd) and a magnetic iron oxide shell containing paramagnetic manganese (Pd/Fe|(nMn)-oxide, *n* = 0.25 and 0.5). Doping the iron oxide lattice with Mn significantly increases magnetic saturation, boosting specific loss power up to 1.7 times compared to that of undoped analogs. Interestingly, higher Mn-content in Pd/Fe|(0.5Mn)-oxide leads to a pronounced Mn outer rim, enhancing the heating efficiency at 346 kHz and 23 mT and contributing to the water exchange on the surface of the paramagnetically doped nanoparticles, resulting in additional *T*_1_ MRI contrast. The enhanced magnetic properties of the hybrid Pd/Fe|Mn-oxide nanoparticles enable effective therapeutic outcomes with injection of only small quantities of the material, offering great potential for effective cancer treatment strategies that combine hyperthermia/thermal ablation with radiotherapy while allowing for real-time monitoring via MRI.

## 1 Introduction

One of the most challenging innovations in medicine lies in multifunctional biocompatible nanoparticles (NPs) that can revolutionize cancer diagnosis and therapy.^1^ Among various applied nanomaterials, magnetic nanoparticles (MNPs) are crucial for biomedical applications, such as drug delivery, cell marking, magnetic hyperthermia/thermal ablation (MH/TA), and magnetic resonance imaging (MRI).^2,3^ For example, one limitation of conventional MNPs in thermal treatments is their low heating capacity, expressed as a specific loss power (SLP). This limitation requires the local injection of large quantities of MNPs.^1^ Although toxicity is not necessarily a concern (FDA-approved magnetic iron oxide NPs (Ferumoxytol) are administered intravenously at doses as high as 510 mg for anemia treatment),^4^ injecting large volumes directly into tumors remains a difficult task. Hence, to fully exploit the therapeutic potential of thermal treatments, it is crucial to design MNPs with high heating efficiency at clinically relevant doses (<10 mg/kg). This would enable the generation of sufficient intratumoral and intralesional temperatures required for sensitization (40–44 °C) or the complete eradication (>60 °C) of cancer tissues.^2,5^ Furthermore, SLP requirements vary depending on the volume of the tumors to be treated. In practice, this means that the concentration of the injected MNPs must be varied to achieve a certain therapeutic temperature increase.^6,7^ Therefore, optimization of the nanomaterials for MH/TA, while keeping an efficient heating power, has become a significant challenge in biomedicine. At the same time, optimized magnetic properties designed for thermal treatments may also lead to improved MR-imaging , as both heating performance and contrast enhancement are directly related to saturation magnetization.^2,8^ These objectives can be achieved by tuning the MNPs parameters, such as size, shape, and magnetocrystalline anisotropy.^1^ Saturation magnetization (M_S_) varies with the size of the MNPs until a threshold size is reached, beyond which the magnetization value plateaus and approaches the bulk magnetization value. Simultaneously, an increase in size alters the balance of magnetic interaction, causing the MNPs to transition from superparamagnetic to single-domain, and eventually, to multidomain regimes.^8^ Conveniently, the MNPs preferred in biomedical applications are sufficiently small to remain in the superparamagnetic regime, where magnetism disappears after removing an applied magnetic field, ensuring colloidal stability and resistance to aggregation.^9,10^ Additionally, it appears that MNPs with a size at the optimal threshold between superparamagnetic- and single-domain behavior are ideal for thermal treatment.^1,11^ Thus, tuning the efficiency of MNPs through size adjustments has limitations. On the other hand, tuning the anisotropy of the MNPs is a very promising alternative strategy to increase M_S_ leading to enhanced heating efficiency and relaxivities (*r*_i_, where, *i* = 1 and 2), which stands for relaxation rate enhancement per concentration of magnetic component—a parameter important for MRI.^1^ Ways to influence the effective anisotropy of MNPs are via changes on shape anisotropy, or on magnetocrystalline anisotropy by doping or synthesizing core–shell MNPs.^12^

In the last years, ferrite nanoparticles with the general formula MFe_2_O_4_ (M = Fe, Mn, Co, Ni) have been in the spotlight due to their potential applications in biomedicine, and to their remarkable magnetic properties, which can be modified by introducing the desired composition of the dopants.^13−15^ An example is an increased saturation magnetization of MnFe_2_O_4_ NPs (110 emu/g) compared to Fe_3_O_4_ NPs of the same size (101 emu/g) due to magnetic engineering of the iron oxide nanocrystal by replacement of Fe^2+^ with Mn^2+^.^16^

Lastly, the nanomedicine paradigm is shifting toward the development of therapeutic agents that can generate accompanying imaging signals. This advancement allows for the visualization of the drug delivery and distribution process, monitoring of therapy, and tracking the fate of NPs over time.^17^ Iron oxide NPs are known for their ability to enhance *T*_2_ MRI contrast. When doped with paramagnetic ions, such as Mn^2+^or Eu^3+^, these MNPs may enable dual *T*_1_/*T*_2_ MRI contrast,^18^ which is more desirable in certain clinical cases. While ultrasmall Fe-oxide NPs (<10 nm) have been reported to also exhibit a *T*_1_-effect,^19^ achieving both high *T*_1_ MRI performance and sufficient magnetization for MH/TA applications typically requires optimization strategies beyond mere size manipulations.^20^ Therefore, engineering of magnetization values via the composition of MNPs is critical for developing sensitive magnetic probes for biomedical applications.^11^

**Figure 1:**
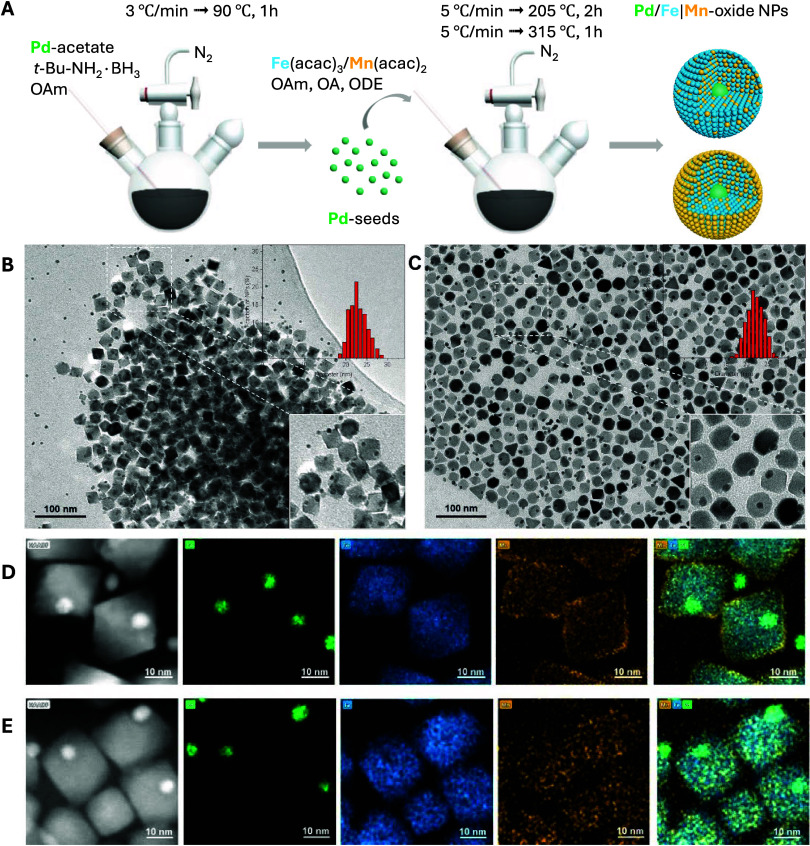
Synthetic procedures for the preparation of Pd/Fe|Mn-oxide nanoparticles (A); TEM images and corresponding size distributions of Pd/Fe|Mn-oxide NPs synthesized with the following precursor ratios: Pd/Fe|(0.5 Mn)-oxide (B) and Pd/Fe|(0.25 Mn)-oxide (C); HR-TEM and EDS elemental mapping with individual signals for Pd (green), Fe (blue), and Mn (orange) of Pd/Fe|(0.5Mn)-oxide (D) and Pd/Fe|(0.25Mn)-oxide (E).

In our previous work, we developed core–shell Pd/Fe-oxide NPs of 20 nm for MR-image assisted MH/TA applications combined with radiotherapy when the ^103^Pd-radioisotope is added to the core.^21−23^ As previously mentioned, tuning the magnetic properties of NPs via their size, while maintaining superparamagnetism, has limitations, while influencing anisotropy presents a valuable alternative. This paper describes the attempt to enhance the magnetic properties of Pd/Fe-oxide NPs by tuning their magnetocrystalline anisotropy through doping of the Fe-oxide coating with Mn. The literature reports the possibility of replacing Fe-cations in the crystal lattice with Mn-cations through the cation-exchange (CE) method,^24^ which involves mixing and heating of the premade Fe-oxide NPs with additional precursors and surfactants. Therefore, in view of the eventual need for ^103^Pd-radiolabeling, the MNPs in this study were synthesized using a seed-mediated thermal decomposition with varying amounts of the Mn-precursor (Figure 1A). The similar sizes and shapes of the obtained Pd/Fe|Mn-oxide NPs allowed us to investigate the effect of Mn-doping on magnetic properties, heating, and imaging performance.

## 2 Materials and Methods

### 2.1 Reagents

Palladium(II) acetate, oleylamine (OAm), *tert*-butylamine-borane complex, ethanol, hexane, iron(III) acetylacetonate (Fe(acac)_3_), manganese(II) acetylacetonate (Mn(acac)_2_), 1,2-hexadecanediol, 1-octadecene (ODE), oleic acid (OA), chloroform, 1,2-distearoyl-*sn*-glycero-3-phosphoethanolamine-*N*-[carboxy(polyethylene glycol)-2000] sodium salt (DSPE-PEG_2000_-COOH), ethylenediaminetetraacetic acid disodium salt dihydrate (EDTA), sodium hydroxide (NaOH), manganese(II) chloride tetrahydrate (MnCl_2_·4H_2_O), toluene, and all chemicals were purchased from Sigma-Aldrich and used without further purification.

### 2.2 Preparation of Oleylamine-Capped Pd NPs (Seeds)

OAm-capped Pd-seeds were prepared based on previously published protocols.^25,26^ Briefly, Pd(II) acetate (56 mg, 0.249 mmol) was added to 15 mL OAm in a 3-neck round-bottom flask. The reaction mixture was heated to 60 °C in 10 min under a stream of nitrogen gas and vigorous stirring. In parallel, the *t*-butylamine-borane complex (130 mg, 1.495 mmol) was dissolved in 3 mL of OAm and injected into the reaction mixture via a septum, once the temperature reached 60 °C. After addition, the reaction mixture was further heated to 90 °C with a heating rate of 3 °C/min and kept at this temperature for 60 min. After that, the reaction mixture was left to cool down to room temperature and the Pd NPs were collected by addition of 30 mL ethanol and centrifugation for 8 min at 10,500 rpm (11,830*g*). The Pd-seeds were stored as such until further use. The final product was redispersible in organic solvents such as toluene, hexane, or chloroform.

### 2.3 Preparation of Pd/Fe|Mn-Oxide via a Seed-Mediated Method

The procedure employed to prepare Pd/Fe|Mn-oxide NPs for this study was adopted from protocols presented in previously published articles.^27,28^ Fe(acac)_3_ (23.5 mg, 0.066 mmol) together with Mn(acac)_2_ (8 mg, 0.033 or 4 mg, 0.015 mmol) and 1,2-hexadecanediol (50 mg) were added to a three-neck round-bottom flask containing 20 mL of ODE, 660 μL of OA, and 65 μL of OAm. OAm-capped Pd NPs (5.3 mg, 0.05 mmol) were dispersed in approximately 0.4 mL hexane and sonicated for 5 min. Next, the Pd-seeds in hexane were added to the 3-neck round-bottom flask. The reaction mixture was slowly heated to 120 °C under a N_2_ flow and vigorous stirring and left at this temperature for 20 min to ensure the complete removal of hexane. Subsequently, the reaction mixture was further heated to 205 °C with a heating rate of 5 °C/min and kept at this temperature for 120 min. After that, the reaction mixture was further heated to 315 °C with a heating rate of 5 °C and kept at this temperature for 60 min. Next, the reaction mixture was left to cool down to room temperature, and the Mn-doped Pd/Fe-oxide NPs with a different Fe:Mn ratio (Pd/Fe|(0.5Mn)-oxide and Pd/Fe|(0.25Mn)-oxide) were collected by addition of 30 mL ethanol and centrifugation at 10,500 rpm (11,830*g*) for 4–6 min. The procedure was repeated several times with ethanol and one or two times with a combination of ethanol and hexane in equal volumes. Lastly, the NPs were dried with a gentle flow of N_2_/compressed air and stored as such until further use. The final product could be redispersed in organic solvents such as toluene, hexane, chloroform.

### 2.4 Preparation of Pd/Fe|Mn-Oxide via the CE-Method

Cation exchange of Mn^2+^ with Fe^2+^ was carried out using a previously published protocol.^29^ Pd/Fe-oxide seeds were first prepared using the above-mentioned method. 40 mg of these NPs was dispersed in 20 mL mixture of ODE and toluene (1:1 v/v), heated to 100 °C, and kept at this temperature for 30 min. After cooling down to room temperature, 9.7 mg (0.4 mmol) MnCl_2_·4H_2_O were added into the flask directly. The mixture was then heated to 180 °C and maintained for 2 h under a N_2_ atmosphere. After the solution was cooled naturally, the Mn-doped Pd/Fe-oxide NPs were collected by centrifugation at 10,500 rpm (11,830*g*) for 4–6 min and washed three times with a mixture of ethanol and hexane in equal volumes. The NPs were dried by a gentle flow of N_2_/compressed air and stored as such until further use. The final product could be redispersed in organic solvents such as toluene, hexane, or chloroform.

### 2.5 Dispersion of Pd/Fe|Mn-Oxide NPs in Aqueous Media with DSPE-PEG_2000_-COOH

The synthesized Pd/Fe|Mn-oxide NPs were transferred into water by means of functionalization with DSPE-PEG_2000_-COOH following a slightly modified protocol presented elsewhere.^30^ First, 2 mg of Pd/Fe|Mn-oxide NPs was dispersed via ultrasonication in 1 mL of CHCl_3_ containing 1.5 mg of PEG surfactant. The vial containing the mixture was left open for 24 *h* for the slow evaporation of the solvent until *a* pasty precipitate remained at the bottom of the vial. The residual solid was heated to 80 °C for 10 min in a vacuum oven and subsequently flushed with a gentle N_2_ flow to ensure complete removal of CHCl_3_. Next, 1 mL of Milli-Q water was added to the precipitate, and the mixture was sonicated for 15 min until a colloidal aqueous suspension was obtained. The colloidal suspension of NPs was pipetted into Eppendorf vials, and the unbound polymer and excess lipids were removed by two rounds of centrifugation for 1 h at 19,600 rpm (30,000*g*) and subsequent removal of the supernatant. Lastly, the NPs were collected in 1–2 mL of Milli-Q water and kept as such for further use.

### 2.6 Pd- and Mn-Leakage Study

Pd/Fe|Mn-oxide NPs (2.5 mg) with the surface covered with DSPE-PEG_2000_-COOH surfactant were resuspended in 0.5 mL of different incubation media (1 mM EDTA (pH 7.4), acidified water (pH 6.5), saline, and serum). The NPs were left in suspension for a specified amount of time (24h for EDTA, and 48h for all other media), after which they were placed in an Amicon Ultra Centrifuge tube with a centrifugal concentrator Ultracel (30 kDa MWCO regenerated cellulose membrane) and centrifuged for 20 min at 4200 rpm. The filtrate was collected and analyzed with ICP-MS to determine the Pd and Mn-content. The NPs were then resuspended in fresh medium and incubated until the next time point, after which the same measurement procedure was applied.

### 2.7 Physical Methods

Particle size, size distribution, and morphology of the samples were determined by transmission electron microscopy (TEM), using a 120 kV Jeol-JEM1400 microscope. The samples were prepared by drop-casting a diluted NP’s suspension in organic solvents such as hexane on a Quantifoil R1.2/1.3 Cu300 grid and evaporating the solvent at room temperature. The mean diameter and the size distribution of the samples were obtained by statistical analysis of around 500–1000 NPs, using TEM images and ImageJ software. The elemental mapping analysis was done with an Oxford Instruments EDS detector X-MAX^N^ 100TLE on the same grids used for TEM. Dynamic light scattering (DLS) was measured on a Malvern Pananalytical Zetasizer Pro (Worcestershire, United Kingdom) equipped with a 633 nm laser.

Magnetic characterization by a superconducting quantum interference device (SQUID) was carried out on an MPMS XL magnetometer from Quantum Design, using about 1–2 mg of dry NPs. The hysteresis loops M(H) obtained under continuously varying applied magnetic fields up to a maximum of ±60 kOe at 5 and 300 K were used for evaluation of saturation magnetization and coercivity. Transmission ^57^Fe Mössbauer spectra were collected at 4.2 K with a conventional constant-acceleration spectrometer using a ^57^Co(Rh) source. Velocity calibration was carried out using α-Fe foil. The Mössbauer spectra were fitted using the Mosswinn 4.0 program. The heating power measurements were performed using the Magnetherm Digital device manufactured by Nanotherics, with either 50 or 60 mm coil, at alternating magnetic fields of 346 or 338 kHz and field strengths of 23 or 18 mT, respectively, used for the calculation of the SLP values. The device was equipped with two glass-fiber optic thermometers (Osensa PRB-G40_2.0-STM-MRI) to measure the temperature at the core (used for SLP) and bottom (to check for a possible precipitation) of the sample. A sample of 1 mg/mL of NPs was inserted in an insulated sample holder to reduce heat loss to the environment and placed in the middle of the coil. The temperature was equilibrated until it varied less than 0.05 °C/min before the start of the measurement. The sample was then exposed to the magnetic field. The heating capacity of MNPs is described as the specific loss power (SLP) or intrinsic loss power (ILP) expressed in W/g_Fe+Mn_ or nHm^2^/kg, respectively.

Measurement of the longitudinal (*T*_1_) and transverse (*T*_2_) relaxation times were performed on a 1.5T at a 450W MR scanner (GE Healthcare, Waukesha, WI) using phantoms prepared in Eppendorf vials by suspending 1 mg of NPs in an agar solution. *T*_1_ relaxation times were measured with an inversion recovery turbo spin echo sequence with the following parameters: repetition time (TR) = 3000 ms, inversion time (TI) = 50, 100, 200, 250, 500, 750, 1000, and 1500 ms, echo time (TE) = 11.2 ms, field of view (FOV) = 192 × 192 mm^2^, slice thickness = 4 mm, acquisition matrix = 192 × 192, and number of excitations (NEX) = 1. The data was analyzed with MATLAB script (R2018b, The MathWorks INC, Natick) developed by Barral et al.^31^ The *T*_2_ relaxation times were measured with a spin echo sequence with the following parameters: TR = 200 ms, TE = 9, 15, 25, 35, 55, and 75 ms, FOV = 192 × 192 mm^2^, slice thickness = 4 mm, acquisition matrix = 128 × 128, and number of excitations (NEX) = 1. The data was fitted with a monoexponential signal decay model using the MATLAB nonlinear curve fitting function. Relaxation measurements were repeated once, and the obtained values were calculated per voxel and reported as the mean with the standard deviation over approximately 120 voxels per sample.

The SLP values and the relaxivities (1/*T_i_*, *i* = 1,2) of the samples were calculated using the concentrations of Fe and Mn obtained from the Inductively Coupled Plasma Optical Emission spectroscopy (ICP-OES) data performed on the same samples after a microwave-aided destruction of NPs dispersed in a 5% solution of HNO_3_. The Electron Paramagnetic Resonance (EPR) spectra were obtained with a Bruker EMXplus X-band EPR spectrometer using a standard resonator (ST9107) at room temperature operating at a microwave frequency of 9.78 GHz, microwave power of 0.002–63 mW (powerplot) or 20 mW, modulation frequency of 100 kHz, and modulation amplitude of 2.5 (powerplot) or 5 G. NPs resuspended in hexane were added to an EPR tube and hexane was evaporated using N_2_ flow. The EPR tube was placed in the cavity, so that only part of the sample was in the measurement window.

## 3 Results and Discussion

### 3.1 Synthesis and Characterization of Pd/Fe|Mn-Oxide NPs

In our previous work, Fe(CO)_5_ was used as the iron precursor for the synthesis of Pd/Fe-oxide NPs to obtain a uniform core–shell structure.^21,22^ However, in this work, Fe(acac)_3_ was considered a better option, as it can be easily adapted to the preparation of MnFe_2_O_4_ by simply adding Mn(acac)_2_ to the reaction mixture along with the Fe(acac)_3_ precursor.^11^ Both Fe(acac)_3_ and Mn(acac)_2_ precursors are readily available, as acetylacetonate group coordinates to a range of metals, including Fe and Mn, with decomposition temperatures around 200 °C.^11,28,32^ The synthesis method used to generate Pd/Fe|Mn-oxide NPs was *a* seed-mediated thermal decomposition, similar to that employed for Pd/Fe-oxide NPs, where premade OAm-capped Pd NPs were introduced into the reaction mixture to act as seeds on which the Fe|Mn-oxide simultaneously nucleates and grows as a coating. However, the newly introduced precursors failed to form core–shell structures, presumably because of the differences in the nucleation/growth regimes of Fe(acac)_3_ and Mn(acac)_2_ as a consequence of their different chemical properties and reactivities. While Fe(CO)_5_ favors heterogeneous nucleation on the seed’s surface followed by successive growth rather than homogeneous nucleation, Fe(acac)_3_ does not exhibit the same preference, with homogeneous nucleation being favored in this case. During the synthesis, this resulted in a mixture of single Fe|Mn-oxide NPs, uncoated Pd-seeds and incomplete core–shell morphology, as reported elsewhere.^27^

Therefore, new measures were employed during the synthesis with Fe(acac)_3_ and Mn(acac)_2_ precursors to promote heterogeneous nucleation and the formation of Pd/Fe|Mn-oxide NPs. These measures included: (i) using smaller amounts of Fe–Mn precursors to avoid supersaturation conditions that promote homogeneous nucleation, (ii) adding less OAm-surfactant during the reaction, (iii) incorporating an intermediate temperature step at approximately 200 °C for an extended period of time to stimulate the formation of Fe(III)-complex with surfactants, (iv) using a long-chain diol (1,2-hexadecanediol) as an accelerant in the formation of Fe–O–Fe bonds and as a mild reducing agent, and (v) increasing the temperature of 315 °C for the growth stage. Similar measures to promote the heterogeneous nucleation of Fe(acac)_3_ on premade seeds have been proposed in other studies.^27^

After tuning the synthetic protocol accordingly, Pd/Fe|Mn-oxide NPs were successfully prepared via thermal decomposition of Fe and Mn acetylacetonate precursors forming a heterogeneous coating on preformed OAm-capped 5 mm Pd-seeds, leading to hybrid MNPs with spherical-squared morphology and sizes around 22 nm, as can be seen in the TEM images presented in Figure 1B,C. Two experiments were conducted, in which different amounts of Mn(acac)_2_ relative to Fe(acac)_3_ were employed during the synthesis: 1[Mn]:2[Fe] and 1[Mn]:4[Fe]. The two batches of NPs will be termed Pd/Fe|(0.5Mn)-oxide and Pd/Fe|(0.25Mn)-oxide, respectively. Independent of the precursor ratio introduced in the reaction, the hybrid MNPs obtained the same spherical-squared morphology (Figure 1B,C). In the bright-field TEM images, the darker areas correspond to Pd, whereas lighter contrast represents Fe|Mn-oxide, as *a* result of the different electron density of Pd compared to Fe and Mn, which both exhibit *a* similar density and cannot be differentiated via different contrast in TEM images, making it impossible to confirm the presence of the two metals in the targeted composition of the hybrid MNPs. For this, EDS elemental mapping analysis was performed on the two batches of synthesized Pd/Fe|Mn-oxide NPs visualizing the individual signals for Pd, Fe, and Mn to confirm their localization within the NP (Figure 1D,E).

Interestingly, in NPs prepared with a higher amount of Mn (Pd/Fe|(0.5Mn)-oxide), the Mn was found to be dispersed within the Fe-oxide coating with most of the Mn atoms located at the surface, forming an outer rim (Figure 1D). This effect was not observed in the particles prepared with a lower Mn amount (Pd/Fe|(0.25Mn)-oxide), most probably due to an insufficient amount of Mn atoms (Figure 1E). The EDS mapping analysis confirmed the successful synthesis of Pd/Fe|Mn-oxide hybrid MNPs, with spherical-square morphologies, relevant sizes (±20 nm), and different amount of Mn-doping, which increased with the higher [Mn]:[Fe] precursor ratio introduced in the synthesis.

**Figure 2:**
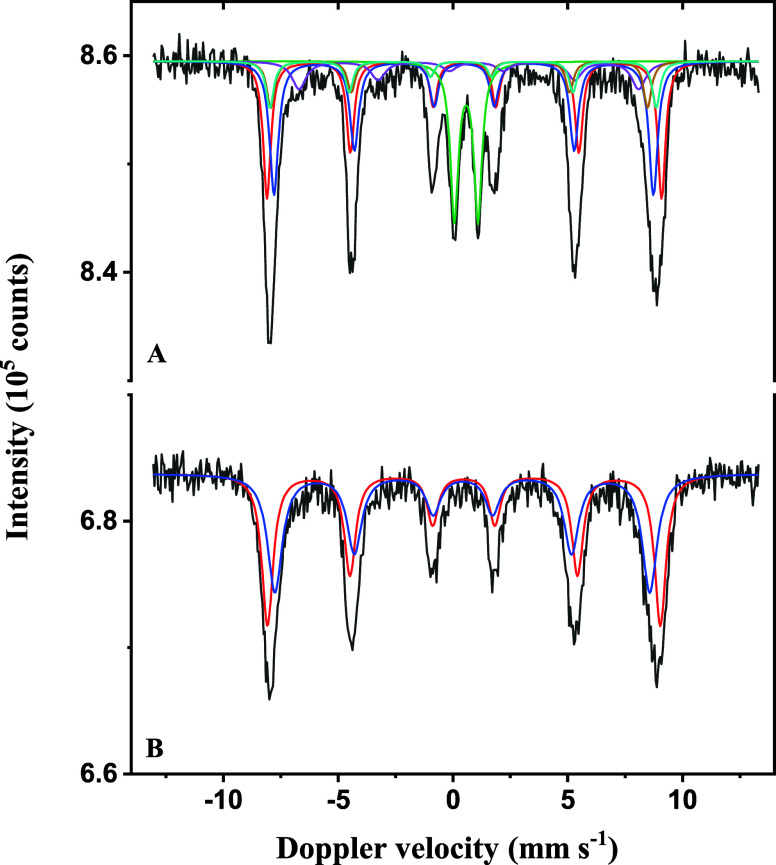
Mössbauer spectra obtained at 4.2 K with the Pd/Fe|(0.25Mn)-oxide (A) and Pd/Fe|(0.5Mn)-oxide NPs (B). The color codes are indicated in Table 1.

To get insight into the effects of Mn-doping on the Fe-oxide framework, which is crucial for interpreting the magnetic properties of the NPs, Mössbauer spectroscopy was performed at 4.2 K, revealing the hyperfine parameters of Fe ions in both Pd/Fe|Mn-oxide NPs (Table 1). The advantage of performing the measurements at this temperature is that magnetite undergoes Verwey transition,^33^ where signals of all three components (tetrahedral Fe^3+^, octahedral Fe^3+^, and octahedral Fe^2+^) can be observed separately, with a typical ratio of 1:1:1. Indeed, this ratio was observed for Pd/Fe|(0.25Mn)-oxide NPs, where 28% of their components could be fitted with contributions from magnetite species. A paramagnetic doublet (16%) observable in the spectrum (Figure 2A) can be explained as the result of the interaction of Fe with paramagnetic Mn dispersed in the lattice without taking part in the crystal structure.

**Table 1:** Mössbauer Fitted Parameters of the Pd/Fe|Mn-Oxide Samples at 4.2 K^a^

sample	IS (mm s^–1^)	QS (mm s^–1^)	hyperfine field (T)	Γ (mm s^–1^)	phase^b^	spectral contribution (%)
Pd/Fe|(0.25Mn)-oxide	0.30	–0.05	51.0	0.41	Fe^3+^ (Fe_3_O_4_, A) yellow	9
	0.40	0.08	52.3	0.41	Fe^3+^ (Fe_3_O_4_, B) cyan	9
	0.85	–0.32	46.0	0.74	Fe^2+^ (Fe_3_O_4_, B) magenta	10
	0.57	1.03		0.42	Fe^3+^ green	16
	0.48	–0.02	51.3	0.46	Fe^3+^ (Fe_3_O_4_, A)^c^ blue	29
	0.50	–0.01	53.4	0.41	Fe^3+^ (Fe_3_O_4_, B)^c^ red	27
Pd/Fe|(0.5 Mn)-oxide	0.42	–0.03	50.7	0.77	Fe^3+^ (Fe_3_O_4_, A)^c^ blue	50
	0.47	0.01	53.1	0.59	Fe^3+^ (Fe_3_O_4_, B)^c^ red	50

a Experimental uncertainties: Isomer shift: IS ± 0.02 mm s^–1^; Quadrupole splitting: QS ± 0.02 mm s^–1^; Line width: Γ ± 0.03 mm s^–1^; Hyperfine field: ± 0.1 T; Spectral contribution: ±3%.

b Tetrahedral (A) and octahedral (B) sites of magnetite, the color code is used in Figure 2.

c Influenced by Mn^2+^ dopants.

Interestingly, another 27 and 29% of the contributions were also fitted with the functions for the tetrahedral and octahedral Fe^3+^ of magnetite, with slight differences in IS and magnetic field due to the influence of Mn and Pd dopants. However, about 28% of octahedral Fe^2+^, typically present in a magnetite spectrum, was absent, likely due to the replacement by Mn^2+^. Since Mössbauer spectroscopy focuses solely on the state of Fe, the effect of Mn interaction was observed indirectly through the exclusive presence of tetrahedral and octahedral Fe^3+^. As Mn^2+^ doping increases, Fe^2+^ ions are progressively replaced, leading to a gradual disappearance of octahedral Fe^2+^ from the spectrum (Figure 2B). A similar pattern was observed in the spectrum of Pd/Fe|(0.5Mn)-oxide NPs, where both tetrahedral and octahedral Fe^3+^ contributed equally (50%), indicating the complete replacement of Fe^2+^. Consequently, an excess of Mn^2+^ accumulated at the surface, forming an outer rim.

**Figure 3:**
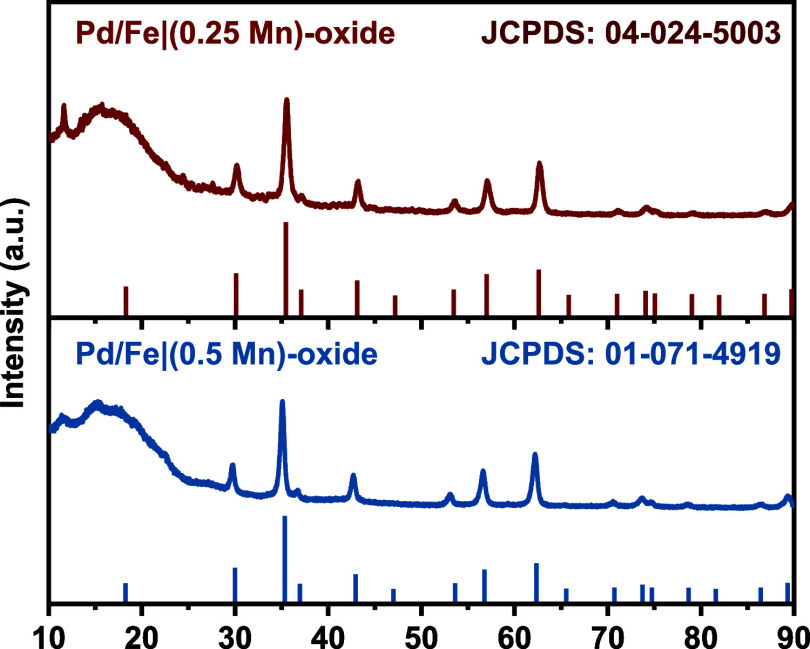
XRD Patterns of Pd/Fe|Mn-oxide NPs.

X-ray diffraction (XRD) performed on both Pd/Fe|(0.25Mn)-oxide and Pd/Fe|(0.5Mn)-oxide NPs further confirmed the appearance of Mn in the lattice by demonstrating cubic phase ferrite, corresponding to the standard card of Fe_2.8_Mn_0.2_O_4_ (JCPDS: 04-024-5003) and Fe_2_MnO_4_ (JCPDS: 01-071-4919), respectively (Figure 3). The average grain size of both nanoparticles was calculated using the Scherrer equation.^34^ The grain size of Pd/Fe|(0.5Mn)-oxide NPs (19.34 nm) is comparable to the overall nanoparticle size, while the grain size of Pd/Fe|(0.25 Mn)-oxide NPs is slightly smaller (14.68 nm). Following the DSPE-PEG_2000_-COOH coating, necessary to transfer the NPs into aqueous media, the morphology remained unchanged (Figure S1). The hydrodynamic size was measured to be 159.4 nm, which is of a similar magnitude compared to that of the undoped Pd/Fe-oxide (113.8 nm) (Figure S2A), and the colloidal stability of the NPs remained good across various buffer solutions and pH lower than physiological (Figure S2B, Table S1).

### 3.2 Magnetic Properties of Pd/Fe|Mn-Oxide MNPs

**Figure 4:**
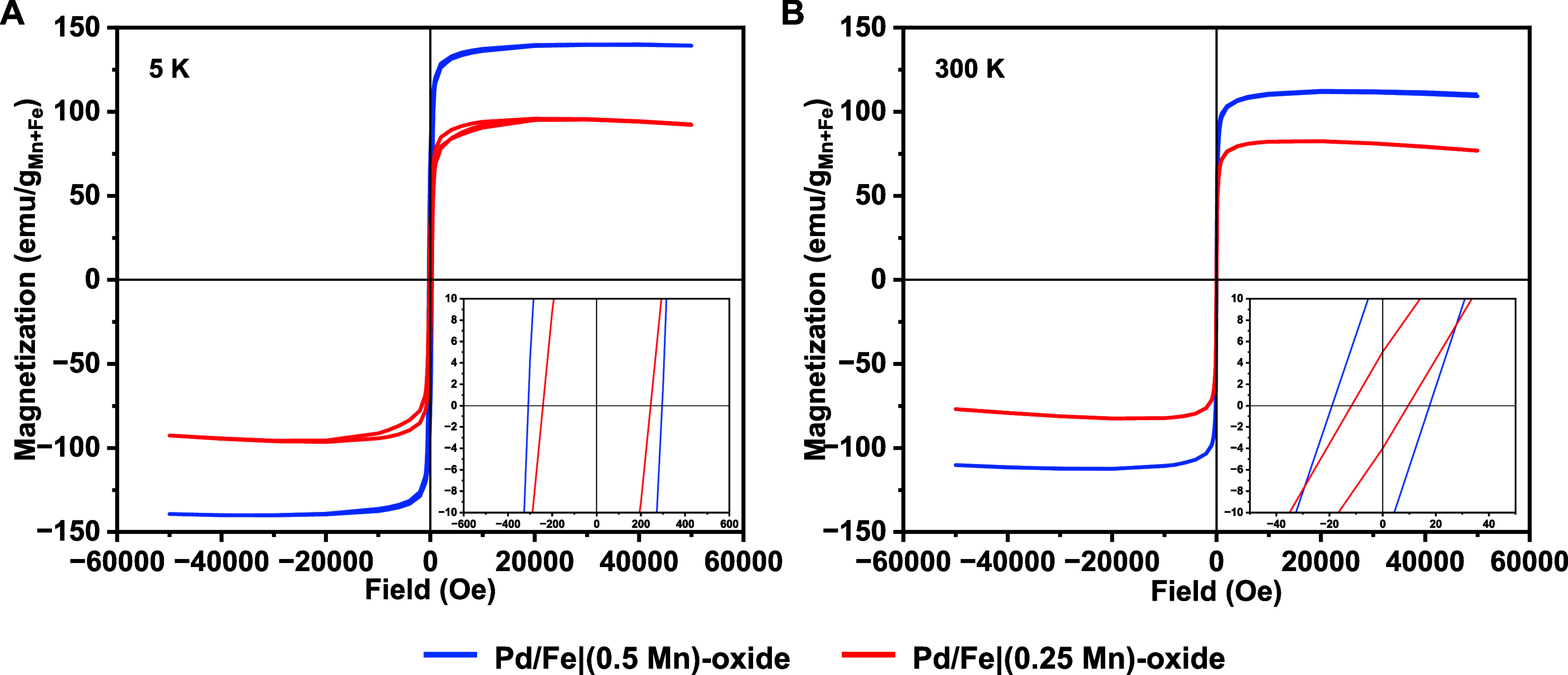
SQUID magnetometry of Pd/Fe|Mn-oxide MNPs: (A) M(H)_T_ curves at *T* = 5 K; (B) M(H)_T_ curves at *T* = 300 K; the insets represent 120′ (A) and 1200′ (B) magnification of the *x*-axis showing coercivities.

The static magnetic behavior of the Pd/Fe|Mn-oxide MNPs was investigated by SQUID magnetometry directly after the synthesis, which means that the MNPs were coated with OA after thermal decomposition and the subsequent cleaning procedure. The magnetization curves resulting from the alignment of the MNPs in the presence of an increasing magnetic field (up to 50 kOe) were measured at 5 and 300 K, as shown in Figure 4A,B, respectively.

The saturation magnetization was determined by extrapolating the highest magnetization values (40 kOe) to the ordinate axis, where the field approaches zero (Table 2). The M_S_ values obtained for the two batches of Pd/Fe|Mn-oxide MNPs were corrected for the presence of Pd-core and organic layer at the surface, hence expressed as emu/g_Fe+Mn_. For both temperatures, 5 and 300 K the saturation magnetization values were higher for the Pd/Fe|(0.5Mn)-oxide, which are NPs with higher Mn-content (Figure 4, Table 2). It is important to note that the hysteresis loops present small dips at high fields because of the strong diamagnetic interaction of the plastic capsule used during the measurements.

**Table 2:** Overview of the Magnetic Properties of Pd/Fe|Mn-Oxide and Pd/Fe-Oxide NPs: Saturation Magnetization (M_S_) at 40 kOe, *T* = 5 and 300 K, Coercivity (H_C_) at *T* = 5 and 300 K, and Calculated SLP, *r*_1_ and *r*_2_ Values

	*M*_S_ (emu/g_Fe+Mn_)		*H*_C_ (O_e_)				
MNPs	5 K	300 K	5 K	300 K	SLP^a^ (W/g_Fe+Mn_)	*r*_1_^b^ (mM_Fe+Mn_^–1^ s^–1^)	*r*_2_^b^ (mM_Fe+Mn_^–1^ s^–1^)
Pd/Fe|(0.5 Mn)-oxide	140	112	308	20	386	8.74 ± 0.3	443
Pd/Fe|(0.25 Mn)-oxide	95	83	240	12	352	5.95 ± 0.4	404
Pd/Fe-oxide^c^	69	61	67	12	233	d	440

a Measured at 346 kHz, 23 mT with STD ≤ 5–10%.

b Measured at 1.5 T and 25 °C.

c From previous work.^21^

d Not measured.

As both batches of MNPs possess similar sizes, the differences in M_S_ values can be attributed to the content of the doping material in their composition. According to multiple literature data, doping Fe-oxide crystal structures with paramagnetic cations, such as Mn or Co, results in an increase in magnetic anisotropy due to the replacement of Fe^2+^/Fe^3+^ cations with more anisotropic Mn^2+^ or Co^2+^. Additionally, the same studies observed that an increased magnetic anisotropy ensured higher M_S_ values, which translates to more effective magnetic properties. As the M_S_ values obtained for Pd/Fe-oxide NPs described previously were not corrected for Pd-core and surfactants,^21^ a relevant comparison could not be made in this case. Nevertheless, it is clear that adjusting the composition of the MNPs is indeed a better strategy to increase the M_S_ of the NPs than size manipulations.

Both magnetization curves at 5 K display hysteresis with substantial coercivity (Table 2), which indicates the typical behavior of superparamagnetic MNPs below their blocking temperature. On the other hand, the MNPs exhibit characteristics of the superparamagnetic state at 300 K, with some low remained coercivity that can be attributed to complex interactions, such as frustrated order or spin canting phenomena. Interestingly, the coercivity values at both 5 and 300 K are also higher for the Pd/Fe|(0.5Mn)-oxide MNPs, which is in line with the increased anisotropy, generally higher for higher Mn-content.^13,24,27^

### 3.3 Pd/Fe|Mn-Oxide MNPs as Hyperthermia/Thermal Ablation Agents

Thermal therapies in oncology include hyperthermia, which involves increasing *in vivo* temperatures to 41–46 °C to sensitize other treatments such as chemo- and radiotherapy, or thermal ablation where the temperatures are >60 °C or exceed at least 46 °C to induce targeted ablation and cell death, potentially replacing surgery.^35^ Two main mechanisms contribute to the dissipation of heat produced by MNPs when exposed to an alternating magnetic field (AMF), depending on their sizes. In larger MNPs, which enter a multidomain regime, heating results from hysteresis losses. In smaller MNPs, like Pd/Fe|Mn-oxide NPs in this study, which exhibit superparamagnetism, the heat production is attributed to Néel and Brownian relaxation mechanisms.^1,5,32^

It is known that magnetic anisotropy plays a critical role in SLP enhancement,^11^ thus the heating efficacy of the two Pd/Fe|Mn-oxide batches with different amounts of Mn-doping was evaluated (Figure S3). The samples containing 1 mg of Pd/Fe|Mn-oxide MNPs dispersed in 1 mL of water were subjected to an alternating magnetic field at the frequency of 346 kHz and *a* field strength of 23 mT. The SLP values were determined for both Pd/Fe|Mn-oxide NPs based on the eq 1:
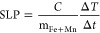
1where, *C* is the heat capacity of the sample, m_Fe+Mn_ is the sum of the masses (g) of Fe and Mn in the colloidal NPs suspension, determined by ICP-OES, and Δ*T*/Δt is the temperature increase measured over time. Compared to the previously reported SLP value for Pd/Fe-oxide NPs of 233 W/g_Fe_,^21^ the SLPs of Pd/Fe|(0.25Mn)-oxide and Pd/Fe|(0.5Mn)-oxide NPs were determined to be 352 and 386 W/g_Fe+Mn_, respectively.

The SLP is directly influenced by extrinsic parameters such as the frequency and amplitude of the applied magnetic field, as well as the viscosity of the surrounding medium.^12^ It is also affected by intrinsic parameters, including the size of the MNPs, their magnetocrystalline or shape anisotropy, and surface functionalization, which alters local magnetization at the surface of the MNPs. Since all of these parameters were consistent across the three investigated MNPs, the superior heating efficiency (increase by 51–66%) of Pd/Fe|Mn-oxide NPs over the Pd/Fe-oxide NPs can be attributed to the presence and amount of Mn in the Fe-oxide coating. This observation aligns with literature reporting that such doping increases crystalline anisotropy leading to enhanced SLP.^1,11,12^

To further investigate the effects of Mn-doping, a batch of premade Pd/Fe-oxide NPs was doped with Mn via a cation exchange procedure^24^ yielding Pd/Fe|Mn-oxide(CE) NPs with spherical-squared morphology and size of 19 nm (Figures S4 and S5). The XRD patterns (Figure S6) confirmed the successful incorporation of Mn into the lattice, showing a highly crystalline cubic phase, corresponding to the standard card of Fe_2_MnO_4_ (JCPDS: 01-071-4919). The heating efficacy of Pd/Fe|Mn-oxide(CE) NPs was compared with that of Pd/Fe|(0.5 Mn)-oxide NPs by calculating their intrinsic loss power (ILP) values (eq 2), which are independent of experimental conditions (i.e., frequency (*f*) and magnetic field (*H*)).
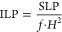
2

The ILP values of CE NPs have previously been shown to be higher than those of Fe NPs due to Mn^2+^ exchange.^29^ However, the ILP value of Pd/Fe|Mn-oxide(CE) NPs (1.289 nHm^2^/kg) was lower than that of the developed Pd/Fe|(0.5Mn)-oxide NPs (3.083 nHm^2^/kg) (Figure S7). This can be attributed to a lower fraction of Fe^2+^ (4%) that has been replaced with Mn^2+^ in the CE-procedure, as revealed by Mössbauer spectroscopy (Figure S8 and Table S2). These results indicate that the seeded growth method is a better choice for Mn^2+^ doping, not only for achieving higher heating efficiency but also because it eliminates the need for an additional synthetic step.

### 3.4 Pd/Fe|Mn-Oxide MNPs as MR Imaging Agents

**Figure 5:**
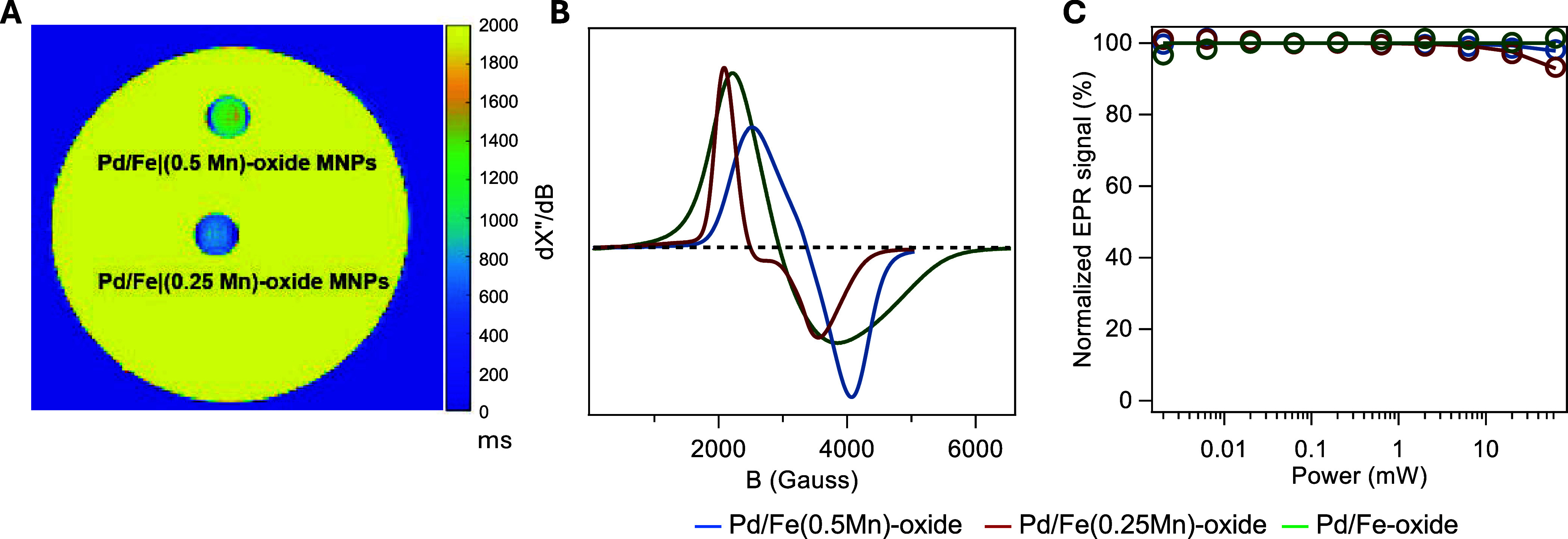
*T*_1_-mapping measured at 1.5 T and 25 °C on agar phantoms containing Pd/Fe|(0.5Mn)-oxide (top) and Pd/Fe|(0.25Mn)-oxide (bottom) NPs (A); EPR spectra of the powder samples, with the signal intensity of the two spectra normalized to their maximal signal amplitude (B); power saturation curves (C). EPR conditions: microwave frequency, 9.78 GHz; microwave power, 20 mW (A) and 0.002–63 mW (B), modulation frequency, 100 kHz, modulation amplitude, 5 G (A) and 2.5 G (B); room temperature.

MRI is a well-known, noninvasive high-resolution imaging technique in medicine that generates anatomical images based on the differences in relaxation of water protons around solid tissue and the surrounding biological media in the presence of magnetic field. The proton relaxation rates can be altered by administration of contrast agents (CAs), enhancing MRI sensitivity and the contrast of the images. MRI CAs function by reducing either the longitudinal (*T*_1_) or the transversal (*T*_2_) relaxation times of protons in the target tissue, generating *T*_1_-weighted images that give positive (bright) image contrast and *T*_2_-weighted images that result in negative (dark) contrast, respectively.^8,11,36^*T*_1_ CAs are typically based on paramagnetic ions like Gd^3+^ and Mn^2+^, often in the form of ion-complexes, whereas *T*_2_ CAs generally rely on superparamagnetic NPs. However, since MNPs designed for MH/TA are intended for intratumoral injection, they are expected to reach much higher local concentrations compared with intravenous administration. At these concentrations, the elevated *r*_2_-values could introduce artifacts in *T*_2_-weighted imaging, limiting its effectiveness until the MNPs diffuse and their local concentration diminishes. By doping Pd/Fe-oxide with paramagnetic Mn, the resulting MNPs may exhibit both *T*_1_ and *T*_2_ contrast effects. To assess this potential dual-contrast capability, *T*_1_ and *T*_2_ measurements were performed on both batches of Pd/Fe|Mn-oxide NPs after transferring them to an aqueous medium with DSPE-PEG_2000_-COOH. Results in Table 2 demonstrate that the *r*_2_-relaxivities of both Pd/Fe|(0.25Mn)-oxide and Pd/Fe|(0.5Mn)-oxide are comparable to those of Pd/Fe-oxide NPs alone. Interestingly, the *r*_1_ value for Pd/Fe|(0.5 Mn)-oxide (8.74 mM_Fe+Mn_ s^–1^) is higher than that for Pd/Fe|(0.25 Mn)-oxide (5.95 m_MFe+Mn_ s^–1^), as expected due to higher Mn-content. Additionally, according to Li et al., *T*_1_-effects are driven by Mn^2+^-ions at the MNPs surface,^37^ which is consistent with our Pd/Fe|(0.5Mn)-oxide NP’s as confirmed by EDS analysis. Figure 5A shows an indication of *T*_1_ contrast enhancement generated by both Pd/Fe|Mn-oxide NPs, with a more substantial effect for Pd/Fe|(0.5 Mn)-oxide. Clearly, the *T*_1_-contrast enhancement produced by the latter MNPs is insufficient and is unlikely to enable them to function as dual *T*_1_/*T*_2_ CAs in practical applications, which would require the *r*_1_/*r*_2_ ratio close to 1. However, understanding how surface Mn-doping affects relaxivity might potentially guide further modifications to optimize contrast characteristics and tune NPs design for other multimodal imaging contexts, where even slight *T*_1_ contributions might enhance overall imaging performance.

To gain more insight into the interplay of the magnetic components constituting the MNPs (i.e., Fe and Mn), EPR spectra of dry Pd/Fe|Mn-oxide NPs with different Mn-content were recorded at room temperature, and the obtained parameters are summarized in Table 3. As shown in Figure 5B, the EPR active spins of both samples resulted in broad spectra ranging from 1500 to 4500 G. The power saturation behavior of the EPR signals in both cases shows almost no saturation, confirming fast relaxation of the spins (Figure 5C). The slight decrease in signal intensity at maximal power (200 mW), being more pronounced for Pd/Fe|(0.25Mn)-oxide NPs, indicates slower relaxation for the less Mn-containing MNPs. This is consistent with the larger Δ*H*_pp_ due to shorter spin lifetime for more Mn-containing NPs, Pd/Fe|(0.5Mn)-oxide. The EPR spectra of the Pd/Fe-oxide NPs with and without Mn-doping obtained at powers corresponding to each data point in the power saturation curves are shown in Figure S9.

**Table 3:** EPR Parameters of Pd/Fe|(0.5Mn)-Oxide and Pd/Fe|(0.25Mn)-Oxide NPs

MNPs	*H*_r_^a^ (mT)	DH_pp_^b^(mT)	*P*_asy_^c^	*g*_peak_^d^	*g*_crossover_^f^	*g*_valley_^g^
Pd/Fe|(0.25 Mn)-oxide	250	147	2.03	3.35	2.44	1.96
Pd/Fe|(0.5 Mn)-oxide	326	155	0.82	2.78	2.07	1.72
Pd/Fe-oxide	295	163	1.86	3.14	2.38	1.81

a Resonance field.

b Line width.

c The ratio of the amplitude of the peak above and below the baseline.

d *g*-factor at the positive peak the EPR signal.

f *g*-factor at H_r_;

g *g*-factor at the negative peak the EPR signal.  with *h* = Planck constant, ν = microwave frequency (9.78 Hz), μB = Bohr magneton, *B* = magnetic field (T). The *g*-factor for a free electron in vacuum *g*_e_ = 2.00232.

The line shape of Pd/Fe|(0.25 Mn)-oxide NPs corresponds to a so-called Dysonian line, which is determined by the diffusion of electrons, the penetration depth of the microwaves, and relaxation time, while the more symmetrical line shape of Pd/Fe|(0.25 Mn)-oxide NPs resembles those observed for superparamagnetic Fe-oxide NPs in general,^38^ as well as the control Pd/Fe-oxide NPs in this study (Figure 5B). According to the Dyson theory, the symmetry parameter *P*_asy_ is correlated with the ratio of electron diffusion and spin lifetime *T*_D_/*T*_spin_. Since the spin lifetime *T*_spin_ (inversely proportional to the Δ*H*_pp_) contains both *T*_1_ and *T*_2_ relaxation in metals, the calculated *P*_asy_ value for Pd/Fe|(0.5Mn)-oxide is significantly lower compared to the less Mn-containing NPs.

### 3.5 Pd- and Mn-Leakage Studies

Since the designed Pd/Fe|Mn-oxide NPs are intended for biomedical applications, their biocompatibility is of great importance. Palladium is considered toxic for the human body, with the hypothesized mechanism being the release of Pd ions, which can elicit a range of cytotoxic effects *in vivo*.^39^ Manganese toxicity (*manganism*) is rare but poses a serious health hazard, potentially leading to severe central nervous system pathologies.^40,41^ The Mn cytotoxicity arises from the triggering of apoptosis in cells accumulating toxic doses of Mn. Depending on the comorbid disease state or dietary variations, bodily efflux and pancreatic elimination may become dysfunctional, potentially affecting the cellular efflux of Mn as well. Changes in glutamate and glutamine metabolism, mitochondrial function, and the triggering of cellular apoptosis and necrosis are key cellular responses to manganism, eventually leading to the neuropsychiatric manifestations of Mn toxicity.^40^ Therefore, with biomedical applications in mind, a study was conducted to determine the amounts of Pd and Mn released from the MNPs. To assess this, Pd/Fe|Mn-oxide NPs functionalized with DSPE-PEG_2000_-COOH were exposed to 1 mM EDTA (a compound typically used in leakage studies as it strongly binds di- and trivalent metal ions),^42^ and incubated for 24 h, 48 h, and 7 d. Since only negligible amounts of Mn and Pd (μg), close to the detection limit and below toxic levels,^39,40^ were found by ICP-OES (Table S3), it could be assumed that Fe-oxide coating encapsulating the Pd-core and the surfactant layers create a sufficient barrier to prevent any Pd or Mn leakage. Similar results were obtained from MNPs incubated with acidified water (pH 6.5), saline, and serum after 48 h (Table S3). Therefore, the Pd/Fe|Mn-oxide NPs can be considered stable for biomedical use.

## 4 Conclusions

In conclusion, this study demonstrates the significant impact of Mn-doping on the magnetocrystalline anisotropy of Pd/Fe-oxide NPs, positioning them as promising candidates for theranostic applications. Using a seed-mediated thermal decomposition method, Pd/Fe|Mn-oxide NPs with varied Mn-doping levels were synthesized while preserving the morphology of their undoped counterparts and thoroughly investigated. The successful incorporation of Mn was confirmed through EDS analysis, while SQUID magnetometry revealed that the Mn-doped NPs are superparamagnetic, displaying high saturation magnetization and low coercivity values. These enhancements translated into superior performance in hyperthermia/thermal ablation therapy, where Mn-doped NPs, especially those with higher Mn-content, exhibited up to 1.7 times greater SLP values under AMF exposure compared with their undoped analogs.

Notably, NPs with higher Mn-content showed significant Mn accumulation on their surface, prompting an investigation of their *T*_1_ MRI contrast properties, alongside their evident *T*_2_ enhancement and demonstrated therapeutic potential. Relaxivity measurements and MRI phantom assessments of the Mn-doped NPs provided with a hydrophilic DSPE-PEG_2000_-COOH layer revealed elevated *r*_1_-values in the samples with the highest Mn-content, reflecting the surface accumulation of Mn. Although the increase in *r*_1_ is insufficient to qualify these NPs as dual *T*_1_/*T*_2_ MRI CAs, these findings emphasize the role of structural modifications in improving both imaging and the therapeutic performance of nanomaterials.

Overall, this work provides valuable insights into the design of multifunctional MNPs as cancer theranostics, offering a promising direction for developing more effective probes for thermal therapy coupled with accurate MRI-based monitoring. This approach is expected to contribute to further advancement of these materials in personalized therapies.

## Supporting Information Available

TEM images of Pd/Fe|Mn-oxide NPs after DSPE-PEG_2000_-COOH coating; DLS data on comparison of the hydrodynamic sizes of Mn-doped and undoped Fe-oxide NPs and Pd/Fe|Mn-oxide NPs dispersed in saline, serum, and acidified water at different time points; comparison of the temperature change within aqueous suspensions of Pd/Fe|(0.5Mn)-oxide and Pd/Fe|(0.25Mn)-oxide NPs; TEM images of Pd/Fe-oxide NPs prepared via thermal decomposition and Pd/Fe|Mn-oxide(CE) NPs prepared via cation exchange; size distribution of Pd/Fe-oxide and Pd/Fe|Mn-oxide(CE) NPs and their XRD patterns; comparison of the temperature change within aqueous suspensions of Pd/Fe|(0.5Mn)-oxide and Pd/Fe|Mn-oxide(CE) NPs; Mössbauer data of Pd/Fe|Mn-oxide(CE) NPs; EPR spectra of Pd/Fe-oxide NPs with and without Mn-doping; Pd and Mn leakage from Pd/Fe|Mn-oxide NPs (PDF)
