# Supplementary material for: Enhancing Magnetic Hyperthermia Efficiency in Pd/Fe-Oxide Hybrid Nanoparticles through Mn-Doping
Source: ACS Appl Nano Mater. 2024 Dec 4;7(23):27465–75. doi: 10.1021/acsanm.4c05452 (PMC11650597; doi:10.1021/acsanm.4c05452)
Supplement: Supplementary file 1 — an4c05452_si_001.pdf [file an4c05452_si_001.pdf]

Supporting Information

Enhancing Magnetic Hyperthermia Efficiency in Pd/Fe-oxide Hybrid Nanoparticles through Mn-Doping

*Alexandra Maier,[1,2,‡] Qi Jia,[1,2,‡] Keshav Shukla,[1,2] Achim Iulian Dugulan,[2] Peter-Leon Hagedoorn,[1] Rogier van Oossanen,[2,3] Gerard van Rhoon,[2,3] Antonia G. Denkova,[2] and Kristina Djanashvili[1,2,]**

[1] Department of Biotechnology, Delft University of Technology, 2628 HZ Delft, The Netherlands
[2] Department of Radiation Science and Technology, Delft University of Technology, 2629 JB Delft, The Netherlands
[3] Department of Radiotherapy, Erasmus MC Cancer Institute, 3008 AE Rotterdam, The Netherlands
*Corresponding author: k.djanashvili@tudelft.nl

Table of Content

Figure S1. TEM images of Pd/Fe|Mn-oxide NPs after DSPE-PEG2000-COOH coating.
Figure S2. DLS spectra showing the hydrodynamic sizes of Mn-doped and undoped Fe-oxide NPs and Pd/Phenoxide NPs dispersed in saline, serum and acidified water.
Table S1. Hydrodynamic sizes of Pd/Fe|Mn-oxide NPs dispersed in saline, serum and acidified water measured by DLS.
Figure S3. Comparison of the temperature change within aqueous suspensions of Pd/Fe|(0.5Mn)-oxide and Pd/Fe|(0.25Mn)-oxide NPs.
Figure S4. TEM images of: (A) Pd/Fe-oxide NPs prepared via thermal decomposition and (B) Pd/Fe|Mn-oxide(CE) NPs prepared via cation exchange.
Figure S5. Size distribution of Pd/Fe-oxide and Pd/Fe|Mn-oxide(CE) NPs.
Figure S6. XRD patterns of Pd/Fe-oxide and Pd/Fe|Mn-oxide(CE) NPs.
Figure S7. Comparison of the temperature change within aqueous suspensions of Pd/Fe|(0.5Mn)-oxide and Pd/Fe|Mn-oxide(CE) NPs.
Figure S8. Mössbauer spectrum obtained at 4.2 K with Pd/Fe|Mn-oxide(CE) NPs.
Table S2. The Mössbauer fitted parameters of the Pd/Fe|Mn-oxide(CE) samples at 4.2 K.
Figure S9. EPR spectra of Pd/Fe-oxide NPs with and without Mn-doping corresponding to each data point in power saturation curve.
Table S3. Pd and Mn leakage from Pd/Fe|Mn-oxide NPs in different buffers and pH conditions.

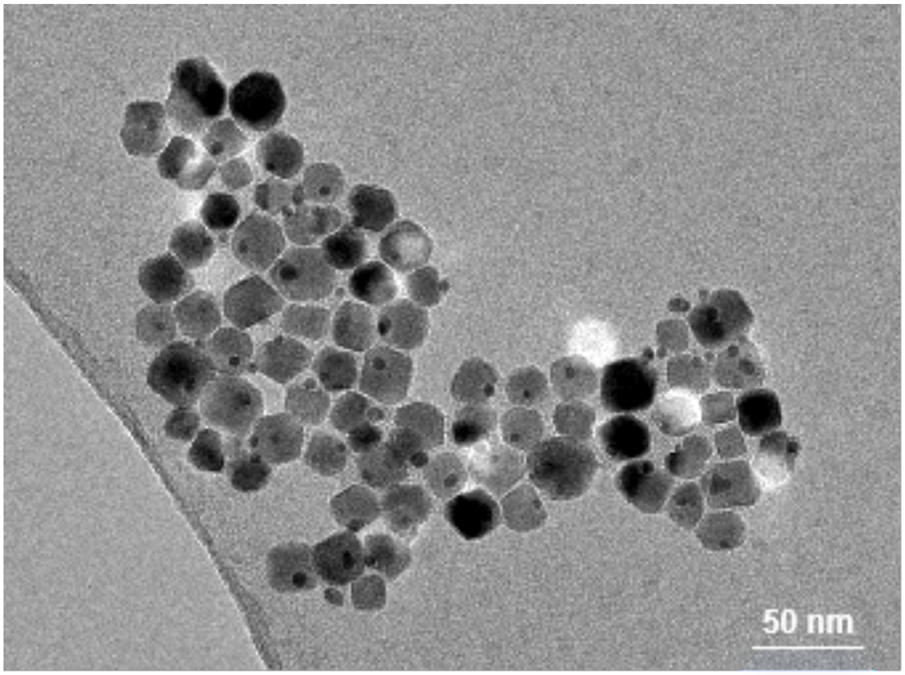

Figure S1. TEM images of Pd/Fe|Mn-oxide NPs after DSPE-PEG_{2000}-COOH coating.

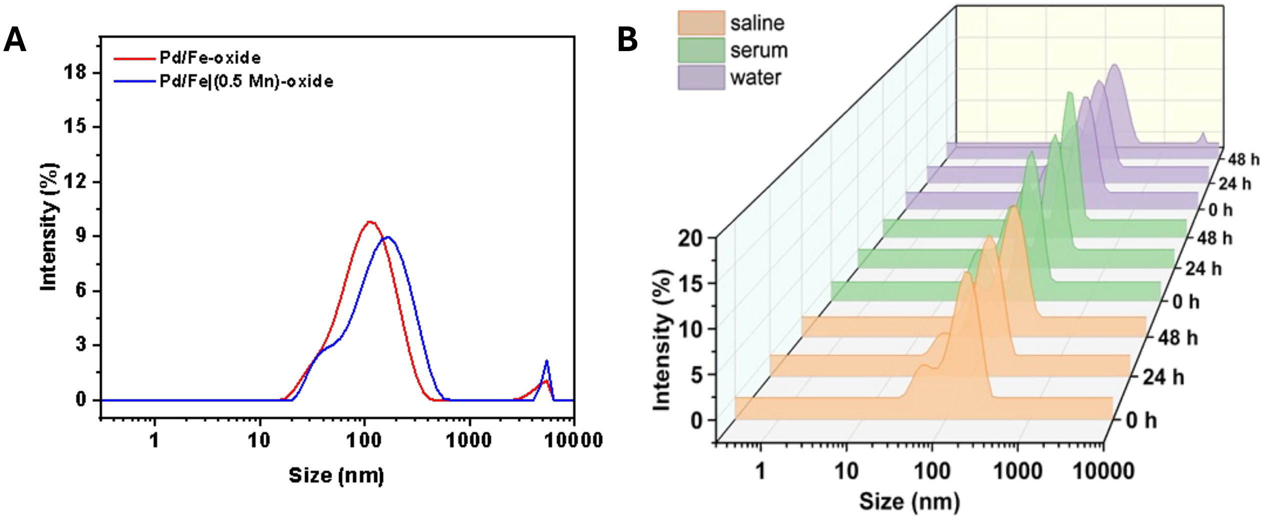

Figure S2. Comparison of the hydrodynamic sizes of Mn-doped and undoped Fe-oxide NPs (**A**). DLS spectra of Pd/Fe|Mn-oxide NPs dispersed in saline, serum and acidified water after 0, 24 and 48h (**B**).

Table S1. Hydrodynamic sizes of Pd/Fe|Mn-oxide NPs dispersed in saline, serum and acidified water after 0, 24 and 48 hours measured by DLS.

Time (h)	Saline		Serum		Water (pH 6.5)	
	Size (nm)	POD	Size (nm)	POD	Size (nm)	POD
0	189.2	0.3316	192.7	0.3566	183	0.3678
24	179.5	0.3188	202.6	0.3719	169.4	0.2987
48	295	167.4	0.3309	197.3	0.3589	188.2

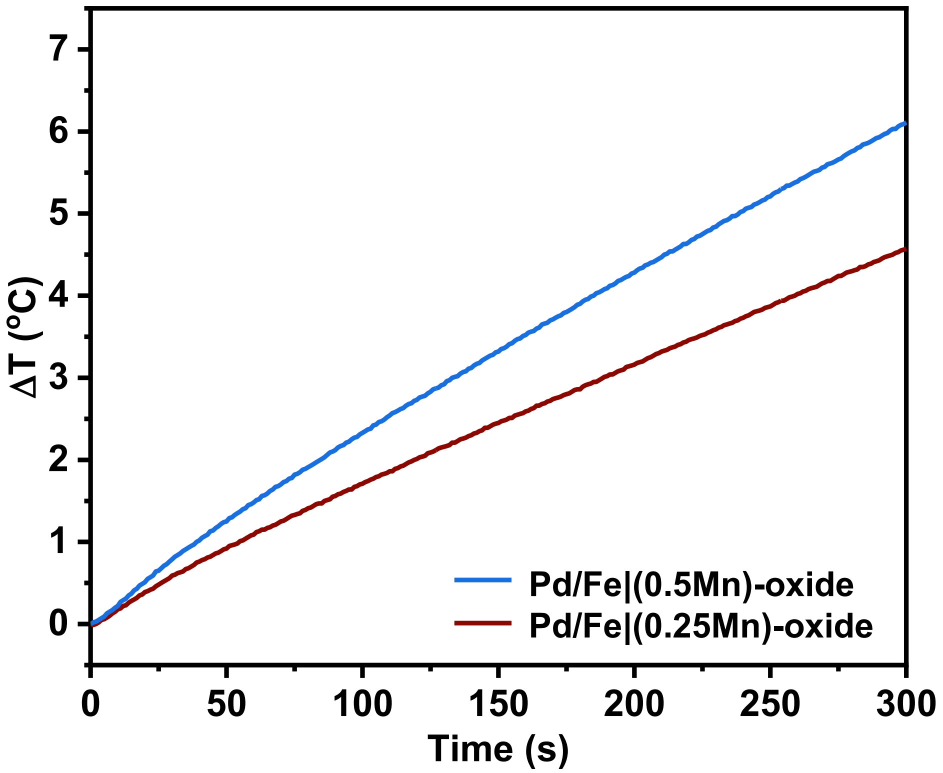

Figure S3. Comparison of the temperature change within aqueous suspensions of Pd/Fe|(0.5Mn)-oxide and Pd/Fe|(0.25Mn)-oxide NPs measured over 6 min.

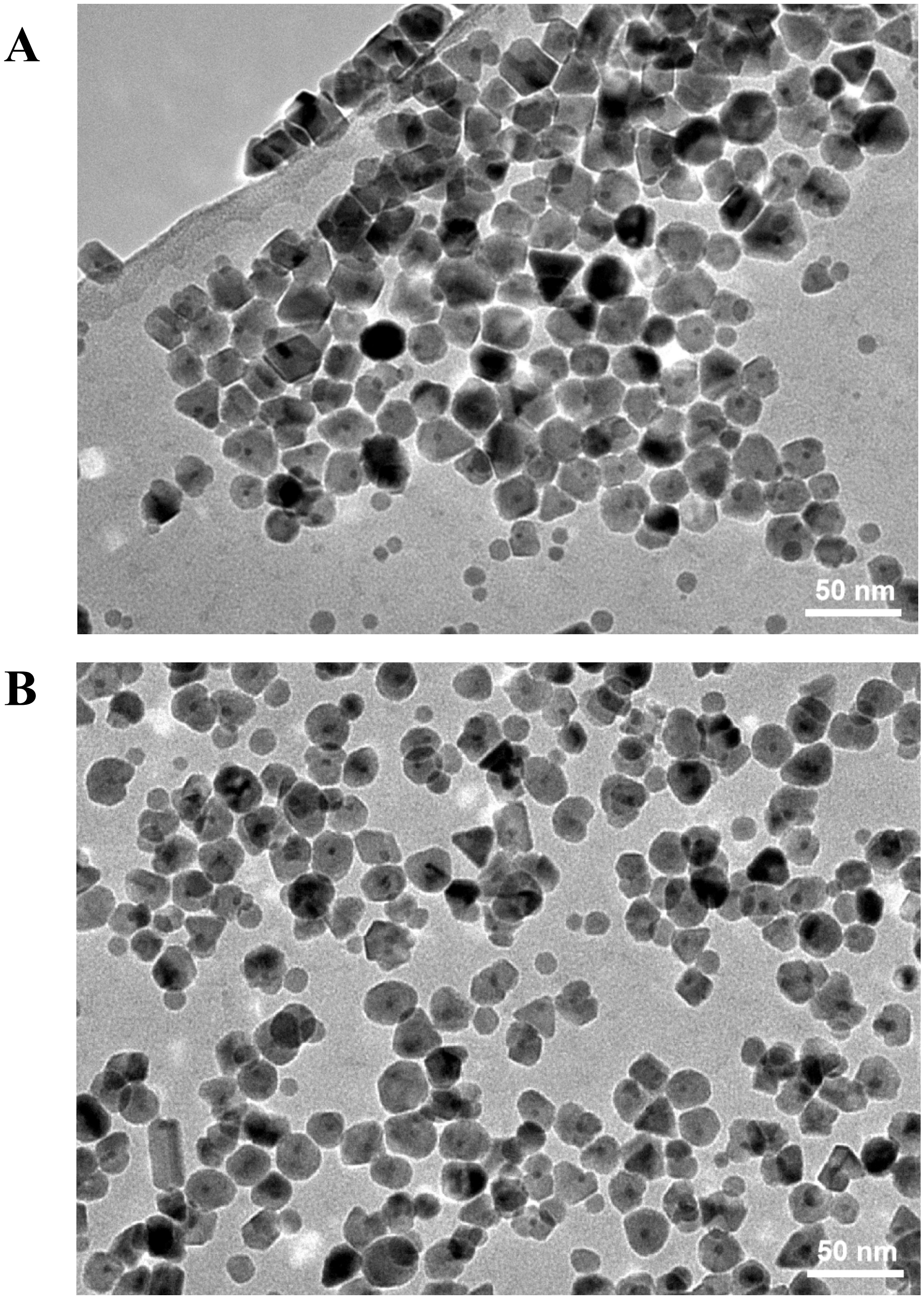

Figure S4. TEM images of: (**A**) Pd/Fe-oxide NPs prepared via thermal decomposition and (**B**) Pd/Fe|Mn-oxide(CE) NPs prepared via cation exchange.

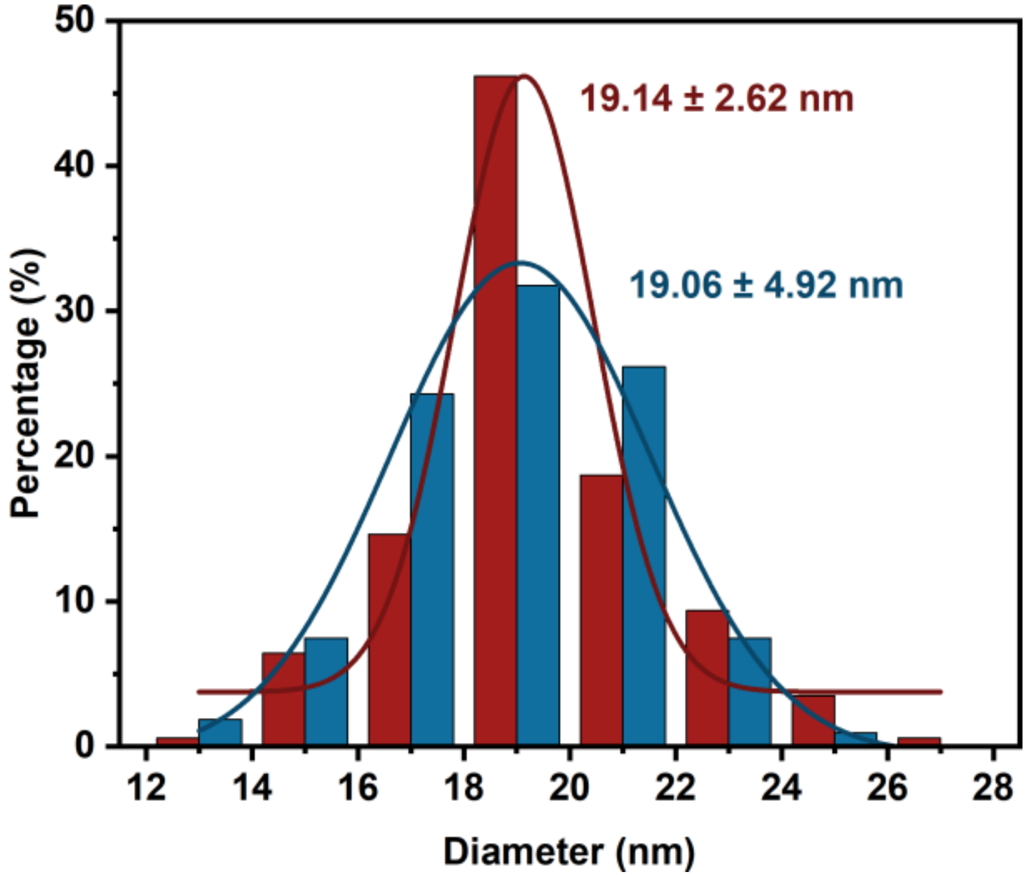

Figure S5. Size distribution of Pd/Fe-oxide (red) and Pd/Fe|Mn-oxide(CE) (blue) NPs.

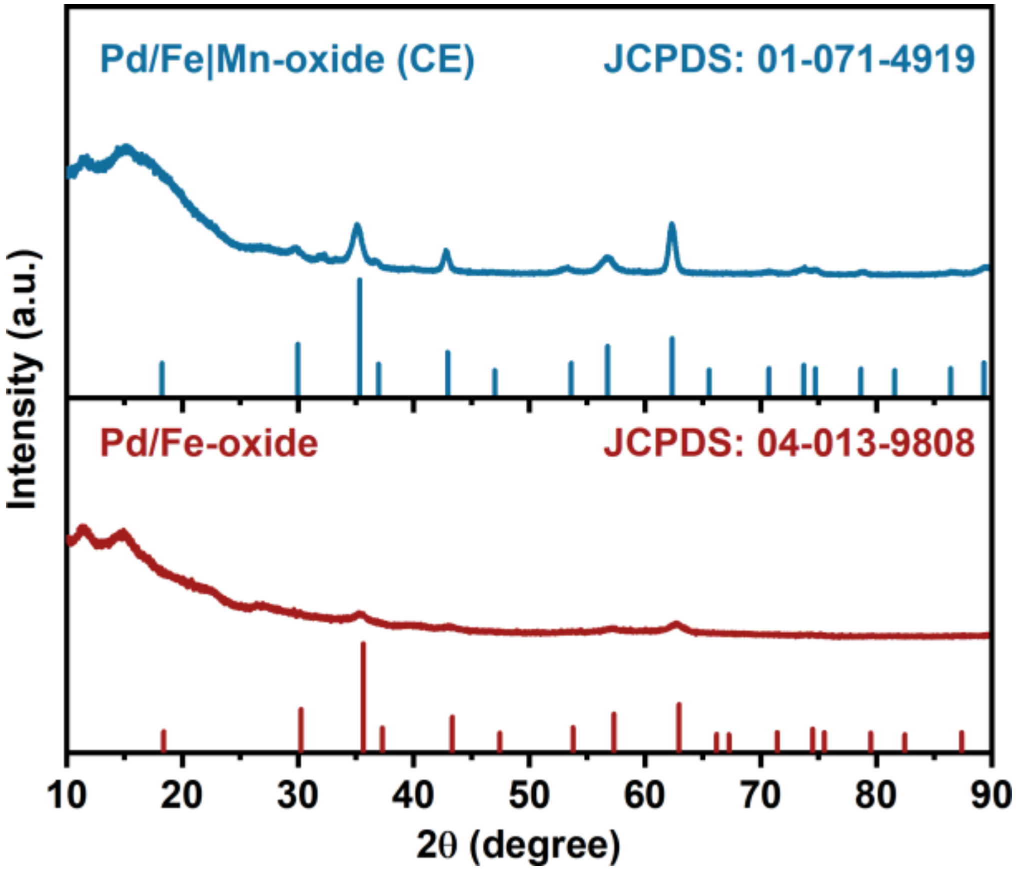

Figure S6. XRD patterns of Pd/Fe-oxide (red) and Pd/Fe|Mn-oxide(CE) (blue) NPs.

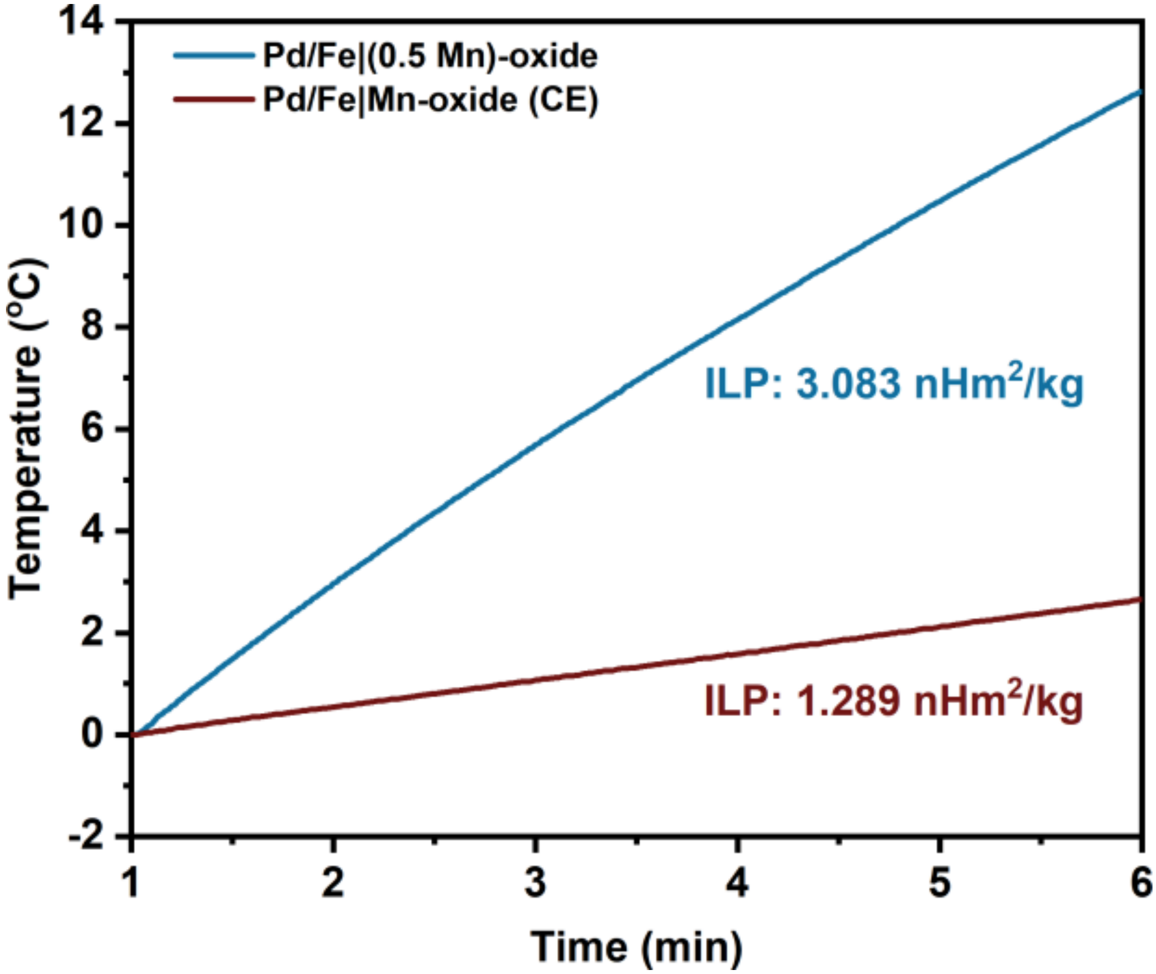

Figure S7. Comparison of the temperature change within aqueous suspensions of Pd/Fe|(0.5Mn)-oxide and Pd/Fe|Mn-oxide(CE) NPs measured over 6 min.

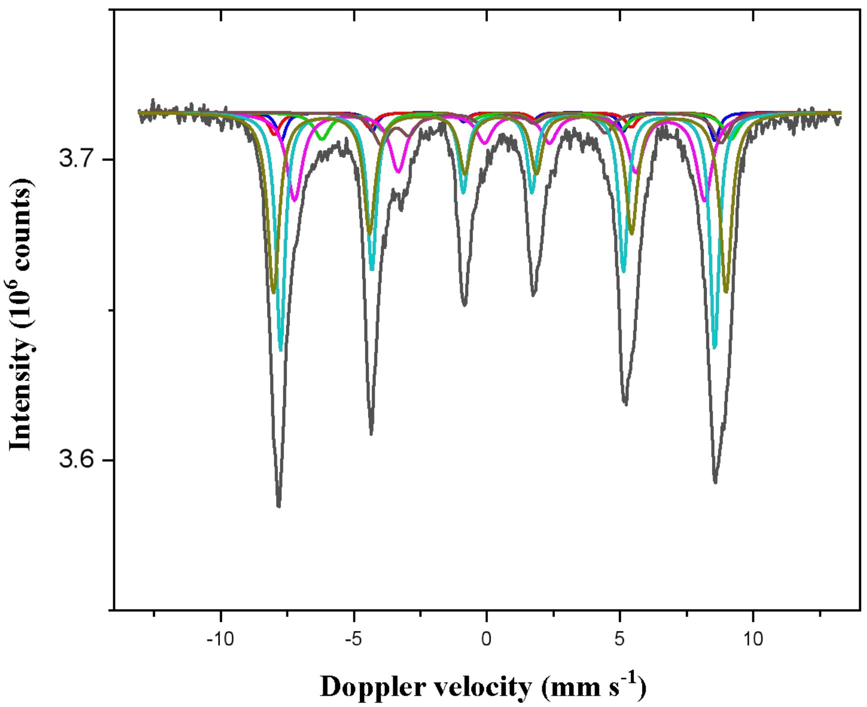

Figure S8. Mössbauer spectrum obtained at 4.2 K with Pd/Fe|Mn-oxide(CE) NPs.

Table S2. The Mössbauer fitted parameters of the Pd/Fe|Mn-oxide(CE) samples at 4.2 K.

IS (mm s^{-1})	QS (mm s^{-1})	Hyperfine field (T)	Γ (mm s^{-1})	Phase [a]	Spectral contribution (%)
0.40	0.01	50.7	0.41	Fe^{3+}(Fe_3O_4,A) dark-yellow	31
0.50	-0.02	52.8	0.54	Fe^{3+} (Fe_3O_4, B1) cyan	30
0.80	-0.63	47.8	0.72	Fe^{2+} (Fe_3O_4, B2) magenta	19
0.94	0.74	46.9	0.72	Fe^{2+} (Fe_3O_4, B3) green	6
1.47	1.50	38.7	0.72	Fe^{2+} (Fe_3O_4, B4) brown	6
0.40	0.01	50.5	0.41	Fe^{3+} (Fe_3O_4, A)* blue	4
0.51	0.00	52.7	0.52	Fe^{3+} (Fe_3O_4, B)* red	4

Experimental uncertainties: Isomer shift: IS ± 0.02 mm s^{-1}; Quadrupole splitting: QS ± 0.02 mm s^{-1}; Line width: Γ ± 0.03 mm s^{-1}; Hyperfine field: ± 0.1T; Spectral contribution: ± 3%; [a]Tetrahedral (A) and octahedral (B) sites of magnetite; *influenced by Mn^{2+} dopants.

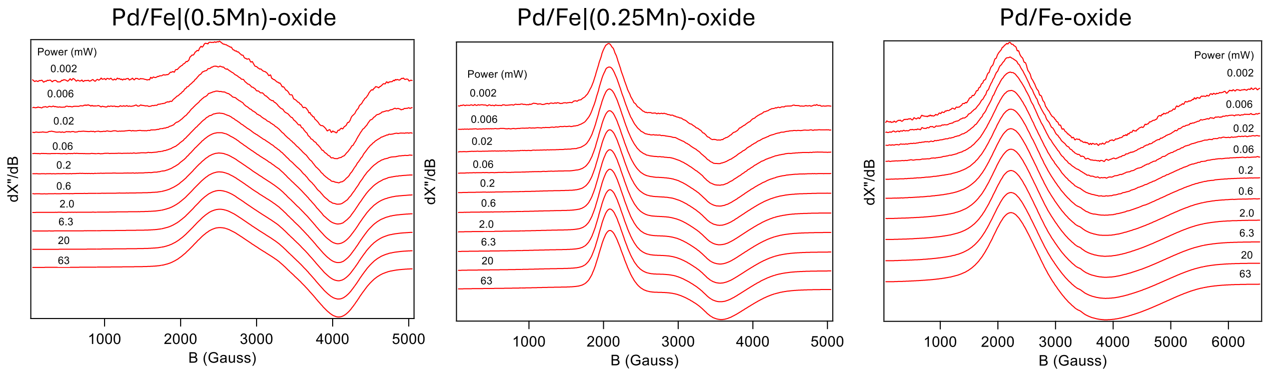

Figure S9. EPR spectra of Pd/Fe-oxide NPs with and without Mn-doping corresponding to each data point in power saturation curve presented in Figure 5C in the main manuscript.

Table S3. Pd and Mn leakage from Pd/Fe|Mn-oxide NPs in different buffers and pH conditions.

Media	Pd		Mn	
	µg	%	µg	%
EDTA (24h)[a]	0.019	0.00081	0.196	0.0081
EDTA (48h)[a]	0.017	0.00071	0.113	0.0047
EDTA (7d)[a]	0.016	0.00066	0.096	0.0039
water (pH 6.5)[b]	0.021	0.00105	0.200	0.00999
saline[b]	0.009	0.00044	0.112	0.00559
serum[b]	0.017	0.00086	0.199	0.00993

[a] pH 7.4; [b] measured at 48h.
